# CPT-11 and concomitant hyperfractionated accelerated radiotherapy induce efficient local control in rectal cancer patients: results from a phase II

**DOI:** 10.1038/sj.bjc.6603322

**Published:** 2006-08-29

**Authors:** V Voelter, A Zouhair, H Vuilleumier, M Matter, H Bouzourene, S Leyvraz, J Bauer, P Coucke, R Stupp

**Affiliations:** 1 Multidisciplinary Oncology Centre, The University of Lausanne Hospitals, Rue du Bugnon 46, CH 1011, Lausanne, Switzerland; 2 Department of Radio-Oncology, The University of Lausanne Hospitals, Lausanne, Switzerland; 3 Department of Surgery, The University of Lausanne Hospitals, Lausanne, Switzerland; 4 Department of Pathology, The University of Lausanne Hospitals, Lausanne, Switzerland

**Keywords:** chemoradiotherapy, CPT-11, distant metastases, neoadjuvant, rectal cancer

## Abstract

Patients with rectal cancer are at high risk of disease recurrence despite neoadjuvant radiochemotherapy with 5-Fluorouracil (5FU), a regimen that is now widely applied. In order to develop a regimen with increased antitumour activity, we previously established the recommended dose of neoadjuvant CPT-11 (three times weekly 90 mg m^−2^) concomitant to hyperfractionated accelerated radiotherapy (HART) followed by surgery within 1 week. Thirty-three patients (20 men) with a locally advanced adenocarcinoma of the rectum were enrolled in this prospective phase II trial (1 cT2, 29 cT3, 3 cT4 and 21 cN+). Median age was 60 years (range 43–75 years). All patients received all three injections of CPT-11 and all but two patients completed radiotherapy as planned. Surgery with total mesorectal excision (TME) was performed within 1 week (range 2–15 days). The preoperative chemoradiotherapy was overall well tolerated, 24% of the patients experienced grade 3 diarrhoea that was easily manageable. At a median follow-up of 2 years no local recurrence occurred, however, nine patients developed distant metastases. The 2-year disease-free survival was 66% (95% confidence interval 0.48–0.83). Neoadjuvant CPT-11 and HART allow for excellent local control; however, distant relapse remains a concern in this patient population.

Patients with rectal cancer are at high risk of both local and distant relapse despite considerable improvements in the multidisciplinary treatment.

Since the introduction of total mesorectal excision (TME) which includes the resection of the entire mesorectal fascia, local recurrences are reported in 5–17% of the patients, depending on the experience of the centre, of the surgeon and whether neoadjuvant treatment has been applied or not (Janjan *et al*, 1998; Kockerling *et al*, 1998; Kapiteijn *et al*, 2001; Glimelius *et al*, 2003; Bosset *et al*, 2005; Engel *et al*, 2005; Gerard *et al*, 2005; Marijnen *et al*, 2005). This is in sharp contrast to former conventional surgery without additional treatment options, which were associated with local recurrence rates of up to 40% (Pahlman and Glimelius, 1990; MacFarlane *et al*, 1993).

In the 1990s, postoperative adjuvant chemoradiotherapy for stage II and III rectal cancer was established based on controlled trials indicating a significant improvement of local control and overall survival (Swedish Rectal Cancer Trial, 1997). Neoadjuvant radiotherapy before surgery was proven to significantly decrease the local recurrence rate and to increase survival using an accelerated and hypofractionated schedule delivering 25 Gy in 5 days (5 × 5 Gy) followed by immediate surgery (Swedish Rectal Cancer Trial, 1997). The delivery of conventional radiotherapy with or without chemotherapy *before* surgery has repeatedly been shown to provide a significant advantage over postoperative, adjuvant treatment in terms of improved local control and reduced toxicity, however, it did not improve overall survival (Pahlman and Glimelius, 1990; Camma *et al*, 2000; Sauer *et al*, 2004). Over the past years, emphasis was put on neoadjuvant chemoradiation followed by optimised surgery. It includes all three modalities – surgery, radiotherapy and chemotherapy, albeit variable in sequence and combination (Swedish Rectal Cancer Trial, 1997; Kapiteijn *et al*, 2001; Bosset *et al*, 2005; Gerard *et al*, 2005).

5-Fluorouracil (5FU) has been the only approved chemotherapy agent for colorectal cancer and had been shown to improve outcome in combination with radiotherapy in rectal cancer (NIH, 1990). Irinotecan (CPT-11), a new chemotherapy agent displaying activity in metastatic colorectal cancer was approved in 1996 as a single agent (Rothenberg *et al*, 1996). In combination with 5FU, it significantly prolongs survival (Saltz *et al*, 2000). Our rationale of integrating an active systemic treatment early in the treatment course of patients with advanced rectal cancer is several-fold. (1) CPT-11 has excellent radiosensitising properties, allowing for better tumour response (Chen *et al*, 1999). (2) The intact vascular bed enables an optimal delivery of the chemotherapy to the primary tumour and (3) the reduction of tumour size improves the resection quality and potentially allows for better organ preservation. (4) Lastly, micrometastases should be eliminated early in the disease course. Indeed, rectal adenocarcinoma has a high invasive potential with up to 60% of patients presenting with systemic metastases (mainly lung and liver), either at initial diagnosis or during follow-up (Tepper *et al*, 2002).

Increased intensity of radiotherapy by hyperfractionation and acceleration (HART) aims at counteracting rapid tumour repopulation. The short treatment time immediately followed by surgery allows for tumour resection before the development of late effects (fibrosis) on normal tissue (Coucke, 1992; Coucke *et al*, 1995, 2006). In head and neck (Saunders *et al*, 1999) as well as in lung cancers (Turrisi *et al*, 1999) HART has shown to improve local control and outcome, and in rectal carcinoma we and others have demonstrated its feasibility (Coucke, 1992; Coucke *et al*, 1995, 2006; Suwinski *et al*, 2006). In line with this hypothesis, the interval between the end of radiotherapy and the surgical procedure has been kept short. The Swedish and the Dutch rectal cancer trials demonstrated excellent local control with a short course of neoadjuvant radiotherapy followed by immediate surgery (Swedish Rectal Cancer Trial, 1997; Kapiteijn *et al*, 2001). However, in particular the latter 5 × 5 Gy radiotherapy regimen is associated with considerable local toxicity (Cedermark *et al*, 1995; Stockholm trial, 1996). With our regimen of hyperfractionated radiotherapy, we aimed at reducing local toxicity while maintaining an intensive short-course regimen.

In a prior phase I trial, we established the neoadjuvant regimen of 3 weekly doses of CPT-11 with concomitant HART followed by immediate surgery (Voelter *et al*, 2003). The regimen demonstrated promising activity in terms of tumour downstaging. Here, we report the outcome of the phase II cohort of 33 patients treated with this regimen.

## PATIENTS AND METHODS

### Patient selection

Patients with locally advanced rectal cancer stage cT3/4 and/or cN+ without evidence of distant metastases were eligible for this trial. Tumours had to be localised below 15 cm from the anal verge as measured by flexible recto- and/or colonoscopy. Normal haematological, hepatic and renal function was required for inclusion. Patients must not have had prior chemotherapy nor radiotherapy and no malignant disease during the past 5 years. All patients gave written informed consent before the start of treatment and the protocol was approved by the local Ethics Committee.

### Treatment schedule

Three doses of CPT-11 at 90 mg m^−2^ were administered as a 30 min infusion on days 1, 8 and 15. Radiotherapy started on day 8, and was delivered at 1.6 Gy per fraction bid (6 h interval) from Monday through Friday for a total dose of 41.6 Gy (26 fractions over 2.5 weeks). Total mesorectal excision was to be performed within 1 week after the end of the neoadjuvant treatment. Postoperative (adjuvant) chemotherapy was planned to start within 4–6 weeks after surgery. The adjuvant treatment consisted of four cycles of CPT-11. As a result of the prior pelvic radiotherapy, escalating doses of CPT-11 starting at 250, 300 and then 350 mg m^−2^, repeated every 3 weeks were recommended.

### Radiotherapy

Patients were irradiated with a linear accelerator using high-energy photon beams of at least 6 MV (Varian Clinac 2100 or Siemens Primus). The dose prescription was at the intersection of the fields (four-field technique). The homogeneity was within 5% of the dose prescribed at the isocentre. All fields were to be treated during each fraction. The field margins were defined according to a ‘standard field’ as described by Gunderson *et al* (1985). The upper limit was the lower level of the fifth lumbar vertebrae and the lateral margins were 1.5 cm lateral to the pelvic bone on both sides. The lower limit included the anal canal only if the tumour was located in the low rectum (0–5 cm form the anal verge). The exclusion of the anal margin was checked by *in vivo* dosimetry using thermoluminescent dosimetry (TLD). Corrections of the lower limit were carried out if required.

### Assessments and statistical considerations

Clinical tumour assessment at diagnosis included computed tomography (CT) of the thorax, abdomen and pelvis as well as magnetic resonance imaging (MRI) of the pelvis and/or transrectal ultrasound of the rectum (TRUS). Tumour downstaging was defined as difference between preoperative cT-stage and postoperative ypT-stage using the sixth edition of the AJCC Cancer Staging Manual. As the clinical staging of N-stage has low specificity and sensitivity using standard staging methods (MRI, TRUS, CT) (Kim *et al*, 1999), the comparison of cN- and pN-stages remains unreliable and was therefore not performed in the present trial.

During the preoperative period patients were followed for toxicity once a week. Physical examination, full blood count and serum chemistry were performed at least once a week. Toxicity was graded according to National Cancer Institute Common Toxicity Criteria (NCI CTC) version 2.0. Postoperative complications were assessed and reported as 30-day postoperative complication rate. After the end of adjuvant chemotherapy, patients were followed clinically every 3 months for the first 2 years, then every 6 months. Radiological evaluation including a CT scan of the chest, abdomen and pelvis, or abdominal ultrasound and chest X-ray were performed every 6 months for at least the first 3 years. Colonoscopy was performed in regular intervals as recommended by the Swiss Society of Gastroenterology.

The primary end point was local recurrence-free survival, with overall survival, patterns of failure and toxicity as secondary endpoints. We hypothesised that the addition of CPT-11 would increase local control rate. A sample size of 30 was required to detect a reduction of the local failure rate from 15 to <5% with a power of 80% and a standard error of 0.05. All analyses were performed in the intent-to-treat population. Survival data were computed according to the Kaplan–Meier method (Kaplan and Meier, 1958) from the date of inclusion until the date of disease recurrence or death.

## RESULTS

### Patients and treatment delivery

Between 2000 and 2005, 33 patients (20 men and 13 women) were enrolled. Median age was 60 years with a range of 43–75 years (Table 1). Clinical tumour characteristics are listed in Table 2 and were as follows: One patient with a cT2 tumour, 29 cT3, 3 cT4; 21 patients had clinical evidence of nodal involvement (cN+). The tumour was located between the anal verge and 15 cm from the anal verge with a median of 7 cm (Table 1).

All but two patients completed chemoradiotherapy as scheduled without dose reduction or treatment delay (Table 1). All patients received the planned three CPT-11 infusions before surgery. Radiotherapy was discontinued early in two patients due to grade 3 diarrhoea after 40 and 28.8 Gy, respectively. The latter patient received postoperatively a complement of irradiation of 20 Gy (2 Gy daily, five fractions per week) to the sacral concavity and surgical tumour bed combined with 5FU 300 mg m^−2^ day^−1^.

### Preoperative chemoradiotherapy

Overall, the neoadjuvant treatment of HART and CPT-11 was well tolerated. Severe toxicities (grade 3) are summarised in Table 3. The majority of patients experienced diarrhoea that was severe in 24%, but usually easily controlled with loperamide and increased fluid intake. Three patients needed temporarily intravenous hydration. Abdominal cramping occurred in 6% of the patients simultaneously to diarrhoea. Nausea and vomiting were rare as well as severe radiation-related local inflammation (e.g. proctitis). Myelosuppression was mild except in one patient who developed short-lived grade 3 neutropenia after the third CPT-11 dose not requiring any delay in radiotherapy or subsequent surgery. Infections 3° occurred in three patients: fever without proven infection and neutropenia 1° (1), *Clostridium difficile* induced diarrhoea (2); however, no event of neutropenic fever was observed.

### Surgery and postoperative complications

All 33 patients underwent surgery. The median time from the end of radiotherapy to surgery was 6 days (range 2–15) (Table 1). Downstaging of the T-stage was achieved in 11 patients (33%) (Table 2). Ten patients (30%) had nodal involvement at pathological analysis (ypN+).

Twenty-one patients (64%) had sphincter-sparing surgery and 12 (36%) needed an abdomino-perineal resection (APR). A protective ileostomy had not been systematically applied at the beginning of the study although strongly recommended. However, according to an increased incidence of anastomotic leakage (AL) in this patient population (21%) (Voelter *et al*, 2003), the protective ileostomy has been introduced as standard procedure for low anterior resection (overall, 15/21patients had an ileostomy). The AL-rate in the present series is 6%. Overall, severe postoperative complications occurred in 27% of the patients. This includes two cases of AL in the 21 patients with low anterior resection (LAR), one of whom did not have an ileostomy. Four patients (12%) developed pelvic abscess without evidence of leakage (two with APR, two with LAR and ileostomy). One patient experienced postoperative pulmonary atelectasia and another patient perioperative sepsis with prolonged abdominal wound healing. One 71-year old patient died 20 days after surgery due to myocardial infarction and subsequent pneumonia with hypoxia. Another patient needed a definitive colostomy 10 months after LAR due to necrosis of the anastomosis without signs of infection. Of 12 patients with APR, 58% experienced prolonged perineal wound healing. Five patients suffered from anorexia during the postoperative period (15%).

Complete resection (R0) was achieved in 26 patients (79%). The positive circumferential resection margin (CRM) rate was 7/33 (21%), however, no local recurrence occurred during a follow-up of 34–72 months, but in four of these patients, disease recurred at a distant site.

### Adjuvant chemotherapy

Twenty-five patients (76%) received adjuvant chemotherapy. At the time the study was conceived, adjuvant 5FU-based chemotherapy was indicated for patients with a postoperative stage III, applicable in our series to only 30% of patients. In fact, only 13 patients received adjuvant CPT-11 monotherapy, six patients received a combination of CPT-11 and 5FU and five patients were treated with adjuvant infusion of 5FU. No patient experienced severe chemotherapy-related complications despite prior pelvic irradiation. Of the eight patients who did not receive adjuvant chemotherapy, one died, one refused and one patient had ypT1 ypN0 stage without indication for further treatment. In five patients with perioperative complications, no adjuvant chemotherapy was prescribed because of the postoperative delay.

### Pattern of relapse

**Figure 1:**
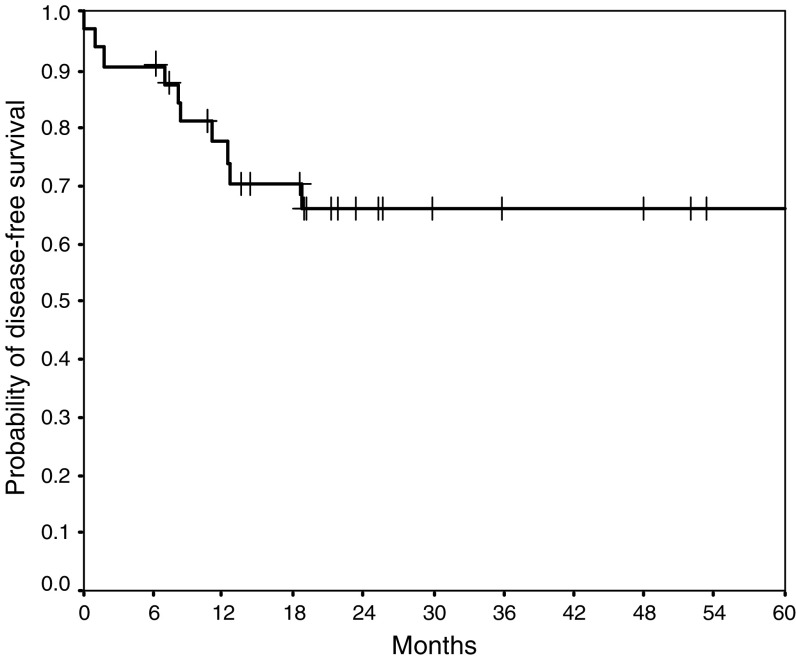
Disease-free survival.

No local recurrence occurred at a median follow-up of 2 years. Distant relapse (metastases) was observed in nine patients (27%), including two of eight patients who did not receive adjuvant chemotherapy and seven of 25 patients who received adjuvant chemotherapy. The disease-free survival at 2 years was 66% (95% confidence interval [CI] 0.48–0.83) (Figure 1). The main distant failure sites were liver and lung (Table 4). One patient was diagnosed with peritoneal metastases during surgery that were resected and the patient is now disease free for 3+ years. The overall survival is 93% (95% CI 0.84–1.0) and the nine patients who developed distant metastases had a 3-year survival of 89% (95% CI 0.79–1.0).

## DISCUSSION

Local failure rates of rectal cancer patients have been substantially reduced with the introduction of more extensive surgery and the use of neoadjuvant chemo- and/or radiotherapy. Relapse rates as low as 5% have been reported with TME alone in high volume centres and with very experienced surgeons (MacFarlane *et al*, 1993). In the Dutch multicentre trial that was also conducted in many community hospitals a local recurrence rate of 11.4% at 5 years with TME alone was observed (Kapiteijn *et al*, 2001; Marijnen *et al*, 2005). This trial confirmed an additional benefit of neoadjuvant short-term radiotherapy even in combination with TME with a reduction of the local relapse rate to only 2.4% at 2 years and 5.8% at 5 years. Others have reported high local recurrence rates up to 17% (Kockerling *et al*, 1998; Camma *et al*, 2000; Colorectal Cancer Collaborative Group, 2001; Glimelius *et al*, 2003; Bosset *et al*, 2005). This discrepancy can be explained by the heterogeneity of the surgery, because TME was not yet widely applied, and by the variable use of 5FU-based chemoradiotherapy. The importance of chemotherapy has been recently underlined by the results of two randomised trials. They demonstrated that 5FU associated to radiotherapy reduced the rate of local failure, at least in patients who had suboptimal surgery because the trials were initiated before TME had been systematically applied (Bosset *et al*, 2005; Gerard *et al*, 2005).

5-Flurouracil has been the mainstay of combined chemoradiotherapy in all large randomised trials (Bosset *et al*, 2004, 2005; Sauer *et al*, 2004). But other active chemotherapeutic agents like CPT-11 are now available and should be investigated within a chemoradiotherapy strategy before surgery.

Here, we demonstrate that CPT-11, given concomitantly with neoadjuvant hyperfractionated accelerated radiotherapy followed by TME is well tolerated and resulted in excellent local control, although median follow-up in our report is currently only 2 years. Purposely, CPT-11 was begun 1 week before the start of radiotherapy, aiming at radiosensitisation by administering the first dose of chemotherapy before starting radiotherapy. Furthermore, chemotherapy needed to be completed approximately 2 weeks before the planned surgery in order to avoid occurrence of myelosuppression in the peri- and postoperative period. Despite a relatively short interval between the end of chemoradiotherapy and surgery, downstaging was achieved in one third of the patients and in almost 80% of patients the resection was complete with negative margins (R0-resection). This compares favourably with other neoadjuvant treatment regimens (Klautke *et al*, 2001; Chau *et al*, 2003; Rodel *et al*, 2003).

The major concern with the use of combined CPT-11 and conventional pelvic radiotherapy has been the occurrence of diarrhoea, a common side effect of both treatments. Our trial suggests that the incidence of severe diarrhoea (24%) is comparable to that in standard 5FU regimens, the latter being associated with diarrhoea in up to 23% of the patients (Table 5) (Minsky *et al*, 1993; Rodel *et al*, 2000; Bosset *et al*, 2004; Sauer *et al*, 2004). Other trials investigating CPT-11 have shown similar results to ours (Minsky *et al*, 1999; Mehta *et al*, 2003; Mitchell *et al*, 2003; Dotor *et al*, 2004; Levine *et al*, 2004; Hofheinz *et al*, 2005; Klautke *et al*, 2005). The severity of diarrhoea is also influenced by the irradiation technique. It has been reported that 20–35% of the patients develop diarrhoea ⩾grade 2 when treated with a conventional radiotherapy technique using large irradiation fields (Minsky *et al*, 1995; Bosset *et al*, 2004).

The overall incidence of postoperative complications is 27% and is in the range of what has been observed with 5FU-based protocols (Swedish Rectal Cancer Trial, 1997; Rodel *et al*, 2000; Ngan *et al*, 2001; Marijnen *et al*, 2002; Mehta *et al*, 2003; Bosset *et al*, 2004). Recently, the large randomised German trial on chemoradiotherapy reported a 36% postoperative complication rate (Table 5) (Sauer *et al*, 2004). The rate of anastomotic leakage (AL) after low anterior resection and TME is usually in the range of 6–18% depending on the series and whether protective ileostomy has been applied or not (Swedish Rectal Cancer Trial, 1997; Rodel *et al*, 2000; Marijnen *et al*, 2002; Mehta *et al*, 2003; Sauer *et al*, 2004). The AL rate of 6% in our trial compares favourably and indicates that the administration of neoadjuvant CPT-11 with radiotherapy is safe.

Delayed perineal wound healing is common after APR and neoadjuvant radiotherapy. It has been described to occur in about 1/3 of the patients (Pahlman and Glimelius, 1990; Kapiteijn *et al*, 2001; Marijnen *et al*, 2002). In the present series, the duration of perineal wound healing was prolonged in more than half of the patients who underwent APR. This is likely due to the use of CPT-11. Ultimately, the wound healed in all seven patients.

Despite the use of neoadjuvant and adjuvant chemotherapy the incidence of distant metastases has not been reduced during the last decades. Distant failure rates of 30–40% are consistently reported throughout all large phase II and III trials and account for the poor outcome of patients with locally advanced rectal cancer compared to patients with colon cancer (Sanfilippo *et al*, 2001; Marijnen *et al*, 2003b; Sauer *et al*, 2004). By 5 years, approximately one third of the patients will die of their disease mainly due to the occurrence of distant metastases (Tepper *et al*, 2002; Bosset *et al*, 2005; Marijnen *et al*, 2005).

In the present trial, a total cumulative dose of neoadjuvant CPT-11 of 270 mg m^−2^ was administered and three quarters of the patients received four cycles of adjuvant chemotherapy. Nevertheless, this did not prevent a 32% incidence of distant metastases. However, by today's standards the patients in this trial may not have received optimal adjuvant therapy. Recently, combination chemotherapy with infusion of 5FU and oxaliplatin has been shown to be superior to infusion of 5FU alone in patients with stage II and III colon cancer (Andre *et al*, 2004), while the combination of 5FU and CPT-11 failed to demonstrate a similar benefit for the combination (Van Cutsem *et al*, 2005). At the time the trial was conceived, adjuvant chemotherapy with bolus 5FU and leucovorin was indicated only for patients with postoperative stage III colorectal cancer, representing only 30% of the patients in the current trial. However, at that time CPT-11 was emerging as a promising new agent with significant activity in second- and first-line therapy of metastatic colorectal cancer. We purposely wanted to expose the patient to the same chemotherapy agent throughout the first-line therapy.

Interestingly, more than half of the patients who had minimal or involved circumferential resection margins consecutively developed distant metastases, while no local recurrence occurred. The majority of these patients had undergone an abdomino-perineal resection. Positive circumferential resection margin involvement is well known to be an important risk factor for disease recurrence, both for distant metastases and/or locally (Nagtegaal *et al*, 2002; Marijnen *et al*, 2003a, 2003b). This seems to be particularly frequent after abdomino-perineal resection (Marijnen *et al*, 2005).

Overall, the association of positive CRM and distant failure suggests that an aggressive biology of the disease leads to early micrometastatic spread while efficient local treatment has prevented local relapse. This is further reinforced by the observation that none of the nine patients who developed distant metastases in this series had a local recurrence at 3 years of follow-up.

These results stress the importance of delivering potent systemic agents early in the treatment course in order to diminish the incidence of distant metastases and to improve the outcome of patients with locally advanced rectal cancer. Future trials should aim at developing regimens with more active chemotherapy including novel targeted agents. In parallel, radiotherapy techniques have to be adapted in order to spare a maximum of surrounding healthy tissue. Intensity modulated radiation therapy is one of these new techniques. For a more conformal approach, a better definition of target volume is a prerequisite. Therefore, MRI and PET-CT have to be evaluated for their role in determining tumour target volumes with high accuracy.

## Figures and Tables

**Table 1:** Patient and treatment characteristics

*Patients and tumour*	
*N*=33	
Male	20
Female	13
	
*Age*	
Median	60 years
Range	43–75 years
	
*Tumour location*	
Median	7 cm
Range	Anal verge – 15 cm
	
*Treatment delivery*	
Radiotherapy completed	31 (94%)
Chemotherapy completed	33 (100%)
Surgery	33 (100%)
	
*Interval radiotherapy – surgery (days)*	
Median	6
Range	2–15

**Table 2:** Clinical (cT) and pathological (ypT) tumour stages

	**cT2**	**cT3**	**cT4**
ypT1		2	
ypT2		7	
ypT3	1	18	2
ypT4		2	1
			
cN+	21		
ypN+	10		

**Table 3:** Toxicity grade 3, 4 during neoadjuvant radiochemotherapy

**Toxicity**	**(%)**
Diarrhoea	24
Infections	9
Abdominal cramping	6
Nausea	3
Cardiovascular*	3
Proctitis	3
Bleeding	0
Neutropenia	3

* Vasovagal syncope in the context of pyelonephritis 2°.

**Table 4:** Distant failure sites

**UPN**	**Liver**	**Lung**	**Lymph node**	**Peritoneal**
23	1			
25	1			
27		1		
29	1		1	1
30		1		
31	1	1		
32				1
35	1			

Abbreviation: UPN, unique patient number.

**Table 5:** Toxicity of neoadjuvant chemo/radiotherapy trials

**Author**	**Drug**	**Diarrhea 3°4° (%)**	**Postop complic (%)**	**AL (%)**
Minsky ‘99 (Minsky *et al*, 1999)	CPT-11	18	—	—
Mehta ‘03 (Mehta *et al*, 2003)	CPT-11, 5-FU	28	38#	6
Mitchell ‘03 (Mitchell *et al*, 2003)	CPT-11, 5-FU	27	—	—
Dotor ‘04 (Dotor *et al*, 2004)	CPT-11, 5-FU	14	—	—
Levine ‘04 (Levine *et al*, 2004)	CPT-11, 5-FU	3	—	—
Hofheinz ‘05 (Hofheinz *et al*, 2005)	CPT-11, CAP	50–75*	47	7
Klautke ‘05 (Klautke *et al*, 2005)	CPT-11, 5-FU	32	14	9
Voelter ‘06	CPT-11	24	27	6
Minsky ‘93 (Minsky *et al*, 1993)	5-FU	17	45	18
Rödel ‘00 (Rodel *et al*, 2000)	5-FU	23	28	18
Sauer ‘04 (Sauer *et al*, 2004)	5-FU	12	36	11
Bosset ‘04 (Bosset *et al*, 2004)	∅/5-FU®	17/34**	22/23	—
Marijnen ‘02 (Marijnen *et al*, 2002)	∅	14**	48	11
SRCT ‘97 (Swedish Rectal Cancer Trial, 1997)	∅	—	44	11

Abbreviations. Postop complic, postoperative complication rate; AL, anastomotic leakage; 5-FU, 5 Fluorouracil; CPT-11, irinotecan; CAP, Capecitabine.

Fields without values correspond to no reported data.

# Excluding three patients with postoperative anaemia.

* Grade 1–2.

** Grade 2.

® Randomised phase III.
